# VIPhyb, an Antagonist of Vasoactive Intestinal Peptide Receptor, Enhances Cellular Antiviral Immunity in Murine Cytomegalovirus Infected Mice

**DOI:** 10.1371/journal.pone.0063381

**Published:** 2013-05-27

**Authors:** Jian-Ming Li, Kasia A. Darlak, Lauren Southerland, Mohammad S. Hossain, David L. Jaye, Cassandra D. Josephson, Hilary Rosenthal, Edmund K. Waller

**Affiliations:** 1 Department of Hematology/Oncology, Winship Cancer Institute, Emory University, Atlanta, Georgia, United States of America; 2 Duke University School of Medicine, Duke University, Durham, North Carolina, United States of America; 3 Department of Pathology and Laboratory Medicine, Winship Cancer Institute, Emory University, Atlanta, Georgia, United States of America; 4 Department of Pathology and Pediatrics, Emory University, Atlanta, Georgia, United States of America; Charité, Campus Benjamin Franklin, Germany

## Abstract

Vasoactive intestinal peptide (VIP) is a neuropeptide hormone that suppresses Th1-mediated cellular immunity. We previously reported that VIP-knockout (VIP-KO) mice have enhanced cellular immune responses and increased survival following murine cytomegalovirus (mCMV) infection in C57BL/6 mice. In this study, we tested whether treatment with a VIP receptor antagonistic peptide protects C57BL/6 and BALB/c mice from mCMV-infection. One week of daily subcutaneous injections of VIPhyb was non-toxic and did not alter frequencies of immune cell subsets in non-infected mice. VIPhyb administration to mCMV-infected C57BL/6 and BALB/c mice markedly enhanced survival, viral clearance, and reduced liver and lung pathology compared with saline-treated controls. The numbers of effector/memory CD8+ T-cells and mature NK cells were increased in VIPhyb-treated mice compared with PBS-treated groups. Pharmacological blockade of VIP-receptor binding or genetic blockade of VIP-signaling prevented the up-regulation of PD-L1 and PD-1 expression on DC and activated CD8^+^ T-cells, respectively, in mCMV-infected mice, and enhanced CD80, CD86, and MHC-II expression on conventional and plasmacytoid DC. VIPhyb-treatment increased type-I IFN synthesis, numbers of IFN-γ- and TNF-α-expressing NK cells and T-cells, and the numbers of mCMV-M45 epitope-peptide-MHC-I tetramer CD8^+^ T-cells following mCMV infection. VIP-treatment lowered the percentage of Treg cells in spleens compared with PBS-treated WT mice following mCMV infection, while significantly decreasing levels of serum VEGF induced by mCMV-infection. The mice in all treated groups exhibited similar levels of anti-mCMV antibody titers. Short-term administration of a VIP-receptor antagonist represents a novel approach to enhance innate and adaptive cellular immunity in a murine model of CMV infection.

## Introduction

Cytomegalovirus (CMV) is a herpes virus that commonly causes asymptomatic infection in immune-competent individuals, with reported rates of seropositivity >50% [1]. Among patients with intact immune systems, cellular and humoral immune responses to infection are robust, with up to 20% of CD8^+^ T-cells directed to a single immune-dominant CMV peptide following primary infection or reactivation of latent CMV infection [2]. CMV has co-evolved with the immune system to limit the extent of adaptive immunity and preserve latent viral reservoirs in epithelial tissues and leukocytes [3], [4]. Murine CMV (mCMV) infection causes immunosuppression through induction of a immature phenotype in dendritic cells (DC), characterized by down-regulation of MHC-I and -II, costimulatory molecules, and reduced production of proinflammatory cytokines [5], and expression of a MHC class-I (MHC-I) decoy that binds to NK cells and inhibits antiviral cytotoxicity [6], [7]. Activation of NK cells is also suppressed by MCMV through expression of several proteins that downregulate expression of NKG2D ligands [8]–[10].

In patients with immune deficiency from HIV [11], allograft recipients treated with immunosuppressive drug therapy [12], or patients with sepsis [13], reactivation of latent CMV infection is also common, and can lead to life-threatening pneumonia or clinical infections also common in the colon, liver or retina [14]. Immunosuppressed patients who fail to mount cellular immune responses or those with dysfunctional effector T-cells [15] may experience multiple episodes of viremia requiring prolonged administration of antiviral drugs with attendant toxicities [16], [17]. While new drugs in development to treat CMV have improved safety profiles, opportunistic CMV infections in the setting of immune-deficiency remain a significant clinical problem, contributing to up to 15% of deaths after allogeneic hematopoietic stem cell transplantation from unrelated donors [18], [19].

Murine model systems of CMV infection have been used to study immune responses to CMV and the interaction between immune-deficiency and infection risk [20]. MCMV has 70% nucleotide homology to human CMV, with a similar genomic organization [21]. Like human CMV infection, mCMV infects the lung, liver and colon of susceptible mice [22], [23]. The lethality of mCMV infection in murine models is increased in the setting of immune deficiency [24] and mCMV infections can be treated by the adoptive transfer of memory CD8^+^ T-cells to infected mice [24]. We have recently shown that mice genetically deficient for vasoactive intestinal polypeptide (VIP) and peptide histidine isoleucine (PHI) are resistant to mCMV infection compared with wild-type (WT) mice, and that mCMV resistance can be adoptively transferred through syngeneic and allogeneic bone marrow transplantation of VIP-knockout (VIP-KO) BM [25], [26].

The resistance of VIP-KO mice to mCMV infection suggests that physiological VIP signaling during viral infection suppresses innate and/or adaptive antiviral immunity [27] in addition to its well-described pleiotropic effects in regulating gastrointestinal function, cardiovascular tone, and behavior [28]. MCMV infection in WT mice causes transient up-regulation of co-inhibitory pathways, and mCMV infected recipients of VIP-KO BM have decreased expression of PD-L1 on DC, lower levels of PD-1 expression on activated T-cells, and increased expression of CD80 and CD86 on DCs [25]. MCMV-infected VIP-KO mice have higher frequencies and increased activation of mCMV peptide MHC-I tetramer^+^ cytolytic CD8^+^ T-cells and lower viral burdens of mCMV compared with WT mice (22). VIP is synthesized by T-cells, antigen presenting cells (APC) and various non-hematolymphoid cells, including neurons. VIP synthesis in neurons and Th2 T-cells is up regulated by multiple factors including injury, apoptosis, and proinflammatory cytokines, IL-1, IL-6 and TNF [29]–[31]. VIP receptors are expressed on T-cells, B-cells, DC, macrophages, neurons, and pituitary and adrenal glands [32]. VIP, as well as the homologous GPCR peptide PACAP, bind VIP receptors and initiates a cAMP-protein kinase A (PKA) transduction pathway [33], including activation of PI3K/PKC, and p38 MAPK [34]–[36]. Culturing murine bone marrow with VIP generates tolerogenic DC that, when adoptively transferred into recipient mice, prevent lethal inflammatory responses to LPS and graft versus host disease (GvHD) [37], and parental administration of VIP generates Treg cells in vivo that suppress immune responses [38] and inhibit function of DC by down-regulating expression of CD80/86 in inflammation [39].

While mCMV infection in VIP-KO mice and syngeneic recipients of VIP-KO BM is of interest as a model system to monitor cellular and humoral immune responses, the results of these experiments cannot be readily translated to clinical practice. Of note, VIP-KO mice lack both VIP and a related peptide, peptide histidine isoleucine (PHI, 37% sequence homology to VIP), so that effects of mCMV infection seen in VIP-KO mice could represent the absence of VIP and/or PHI signaling, or the remaining activity of a third related peptide, pituitary adenylate cyclase activating polypeptide (PACAP, PACAP27 exhibit 68% sequence homology to VIP), which is not deleted in VIP-KO mice [40], [41].

Recognizing the role of VIP and related peptides in regulating immune responses, we explored, in the current study, whether a peptide antagonist to VIP receptor binding could recapitulate the resistance to mCMV infection seen in VIP-KO mice. Peptides with modified VIP sequences have been developed as tools to study VIP signaling. Removing C-terminal amino acid residues of 1–10 in VIP leads to significantly less binding than the full-length 1–28 peptide [42]. Replacement of the first 6 C-terminal amino acids of VIP with the sequence of neurotensin creates a VIP antagonist peptide, VIPhyb [43], that antagonizes VIP, PHI and PACAP binding to human and mouse cells [44]. VIPhyb causes a half-maximal inhibition of VIP binding to VIP receptors on lymphocytes at 5 µM, and maximal inhibition of VIP-induced cAMP generation at 10 µM [45] in experimental conditions. VIPhyb was selected for these studies as if has been previously administered to adult mice without adverse effects and has less neurotoxicity than other VIP peptide antagonists that inhibit VIP binding more potently [43]. Our results show that survival, viral clearance, and adaptive cellular immune responses following mCMV infection are significantly enhanced by administration of a short course of daily treatment with a small molecule antagonist of VIP receptor.

## Materials and Methods

### Mice

Two strains of male mice were used: C57BL/6 (H-2K^b^, mCMV-resistant, Ly-49H^+^, Th1-polarized) and BALB/c mice (H-2K^d^, mCMV susceptible, Ly-49H^−^, and Th2 polarized) [46], [47]. Vasoactive intestinal peptide/peptide histidine isoleucine (VIP/PHI)-KO mice (VIP-KO), congenic to C57BL/6 mice have been previously described [48]. Syngeneic WT littermates of VIP-KO were used for controls. BALB/c mice were purchased from JAX Lab. All of the other mice were 8–10 weeks old and bred at the Emory University Animal Care Facility (Atlanta, GA). Experimental procedures conformed to *the Guide for the Care and Use of Laboratory Animals*, and were approved by the Emory University Institutional Animal Care and Use Committee (IACUC).

### VIPhyb Administration and mCMV Infection

WT mice were treated with daily subcutaneous injections of VIPhyb (H2N-KPRRPYTDNYTRKQMAVKKYLNSILN-amide, New England Peptide, Garder, MA. 10 µg/100µL per mouse) for 1 week starting the day prior to infection with the Smith strain of mCMV that had been passaged in vivo in salivary glands and frozen in aliquots in liquid nitrogen. The LD50 of mCMV for BALB/c mice (mCMV sensitive) is 2×10^4^ plaque-forming unit (PFU); the LD50 for C57BL/6 mice (mCMV resistant) is 8×10^5^ PFU [49], [50]. WT and VIP-KO mice were given either low dose (4×10^3^ PFU for BALB/c; 1×10^5^ PFU for B6), or high dose (5×10^4^ PFU for BALB/c; 1×10^6^ PFU for B6 mice) by intraperitoneal injection. Mice were monitored for signs of illness including hunched posture, decreased activity, weight loss, and survival, and euthanized if moribund or experienced ≥25% weight loss or clinical score >5.

### Analysis of Peripheral Blood and Spleen Samples

Blood and spleens were obtained at necropsy 40 hours [51], 3, 7, 10, 14, and 17 days after mCMV infection. Leukocytes, red blood cells and platelets were counted using a Beckman Coulter automated counter. Blood and spleen samples were depleted of red blood cells by ammonium chloride lysis and washed twice. NK, NK-T, and T-cell subsets were enumerated using CD3 PE/PE-Cy7/FITC, CD4 PE-Alexa610/PE-Alexa700, CD8 PE-Cy7/Per-CP, CD62L FITC/APC, CD25 APC-Cy7, CD69 PE-Cy7, KLRG1 APC, PD-1 PE, FoxP3 and NK1.1 PE (Pharmingen). APC labeled mCMV M45 aa-985∼993- peptide-HGIRNASFI-H-2D^b^ tetramer was obtained from the Emory Tetramer Core Facility. All samples were analyzed on a FACS Canto (Beckon Dickinson, San Jose, CA) and list mode files were analyzed using FlowJo software (Tree Star, Inc. 2007). Samples for flow cytometric analysis of mCMV-M45 epitope peptide-MHC-I tetramer^+^ CD8^+^ T-cells (tetramer^+^ CD8^+^ T-cells) were gated for lymphocytes in the area of FSC and SSC, using a gate setting for tetramer^+^ CD8^+^ T-cells such that 99.99% of control (non-immune) CD8^+^ T-cells were negative [50]. Flow cytometric analyses of the T-cell markers PD-1, ICOS, CD62L, CD25 and CD69, intracellular cytokines (IFN-γ and TNF-α), and DC markers (I-A^b^ or I-A^d^, CD80, and PD-L1) were analyzed as previously described [25], [52], [53].

### Determination of Viral Load

Viral loads were analyzed as previously described [49]. Briefly, livers were collected from CMV-infected recipients at different time-points, homogenized and stored in −80°C. After thawing, the tissue homogenized were sonicated and centrifuged (10,000 rpm, 10 min, 4°C). Serially diluted supernatants were added to 3T3 confluent monolayers in 24-well tissue culture plates and incubated for 90 minutes at 37°C and 5% CO2, then over layered with 1 mL 2.5% methylcellulose in DMEM (10% fetal calf serum). After 4 days, the methylcellulose was removed and the 3T3 confluent monolayers were stained with methylene blue. MCMV plaques were directly counted under a light microscope (Nikon, Melville, New York) and the total PFUs in each organ were calculated.

### Histopathological Analysis and Scoring

Livers and lungs were obtained at necropsy 3, 7 and 10 days following mCMV infection, fixed in 10% formalin and embedded with paraffin. Five-micrometer sections (5 µM) were sliced onto slides, deparaffinized and stained with hematoxylin and eosin. The numbers of intranuclear inclusion bodies in 20 × magnification fields, infiltrated nucleated cells, necrotic foci in 10 × magnification fields were counted by two pathologists who were blinded to the experiment groups using Olympus BX51 Fluorescence Microscope. Data were averaged from 10 random representative area of 0.26 mM^2^ from 2 sections per mouse and per time-point.

### Measurement of Serum Anti-mCMV Antibody Titers

Sera were screened for antibodies to mCMV by using a commercial ELISA kit (Charles river, Wilmington, MA) according to the manufacturer’s instructions [54]. In brief, the presence of mCMV antibodies in mouse sera were tested using antigen coated 96-well plates Day 0, Day 2, Day 7, Day 10 and Day 17 post infection. Each serum sample was diluted at 1∶3000 (1∶60 for d0 and d2 samples) initially then further diluted to 1∶6000 or 1∶12000 if value exceeded the range of the high positive control sample. Each sample was tested on the partially purified mCMV antigen coated plate and tissue coated well. The antibody titer for each sample was calculated from line-regression with OD_control_ versus concentrations of given control in a 2 fold serial dilutions and then × dilution factor.

### Measurement of Serum Growth Factors, Cytokines, and Chemokines

Serum levels of hematopoietic growth factors (GM-CSF, G-CSF, and IL-3), cytokines (IL-1α&β, IL-2, IL-4, IL-5, IL-6, IL-10, IL-12, IL-13, IL-17, IL-23, IFN-α, β, γ, TGF-β, and TNFα), and chemokines (CCL2, CCL3, CCL5, CCL7, and CCL11; CXCL1 and CXCL10) were measured by Yael Rosenberg-Hasson at University of Stanford with a commercial Luminex kit (Affymetrix, Santa Clara, CA) according to the manufacturer’s instructions.

### In vivo Killing Assay

Naive splenocytes were harvested from CD45.1^+^/CD45.2^+^ heterozygous C57BL/6 mice and pulsed with 3 µM mCMV M45 aa-985∼993- HGIRNASFI peptide in 3% FBS-RPMI 1640 for 90 min at 37°C, and washed three times with ice-cold media. The equal parts of pulsed target splenocytes and non-pulsed splenocytes from CD45.1^+^ B6 congenic mice were mixed together. 40×10^6^ target cells per mouse were injected i.v. Into C57BL/6 CD45.2^+^ VIP-KO mice, VIPhyb or PBS-treated WT littermates that had been infected 9 days earlier with low-dose mCMV, or injected into non-infected WT control mice. Sixteen hours following injection of target cells, recipients were sacrificed, splenocytes harvested, and the numbers of pulsed CD45.1^+^/CD45.2^+^ and non-pulsed CD45.1^+^ target cells quantified by FACS analysis. The specific in vivo anti-viral lytic activity for individual mouse was calculated by the formula: (1- (ratio of immune killing pulsed-target cells/ratio of immune killing non- pulsed target cells)) × 100.

### Statistical Analysis

Survival differences among groups were calculated with the Kaplan-Meier log-rank test in a pair wise fashion. Differences in tetramer response, cytokine levels, DC, and T-cell numbers were compared by 1-way analysis of variance. The data collected from pathological screen were analyzed by nonparametric test. All tests were performed by SPSS version19.

## Results

### Treatment with Peptide Antagonist of VIP Receptor was Non-toxic and did not Alter Numbers of Immune Cells in Non-infected Mice

**Figure 1:**
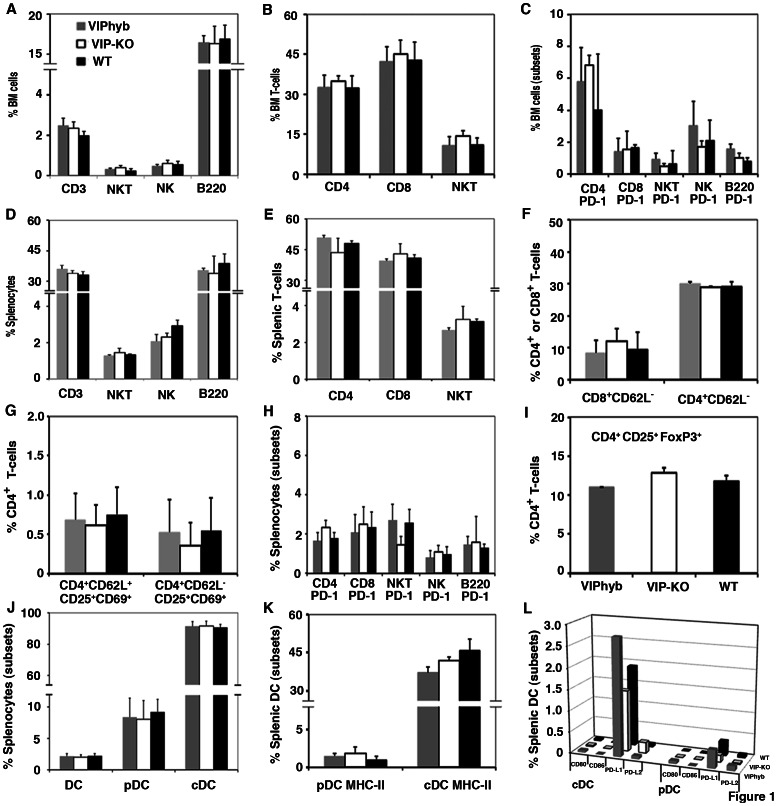
Blocking VIP-signaling did not change the numbers of immune cells. VIP-KO (open bar) mice or WT mice treated with a short-course of VIPhyb (10 µg/mouse s.c., gray filled bar) or PBS (100 µL/mouse s.c., black filled bar) (n = 5–10 per group each experiment) were euthanized one day after the last dose of administration, the spleens, femurs and tibias were isolated. Hematopoietic cells harvested from the spleens and bones were phenotyped by flow cytometry. Data (mean ± SD, n = 25) are summarized from 3 replicate experiments. A–C: Lymphocyte subsets from BM. D–I: Lymphocyte subsets from spleen. J–L: DC subsets from spleen. Data shown on panel L are mean values (SD were 20–30% of the mean values, and not shown for the sake of clarity in the panel).

First, the toxicity of VIPhyb was tested in non-infected C57BL/6 mice. After seven daily sub-cutaneous doses of 10 µg VIPhyb, scores of activity, body weight, fur texture, posture and skin integrity did not differ between VIP-KO, VIPhyb-treated, or PBS-treated mice and remained normal over the following 100 days. Administration of VIPhyb had no significant effect on complete blood count (**data not shown**), bone marrow cellularity or immune cell subsets (B-cells, CD4^+^ T-cells, CD8^+^ T-cells, Treg, NK T-cells, NK cells, cDC and pDC), or their activation status (CD62L, CD25, CD69, PD-1, and MHC-II expression) isolated from bone marrow (**Fig. 1A–C**) or spleen (**Fig. 1D–K**). Of note, less than 3% of DC subsets in the spleens from all of the treated mice expressed CD80, CD86, PD-L1 or PD-L2 **(** **Fig. 1L** **)**. To further exclude effect of residual PACAP signaling on numbers of immune cells in VIP-KO mice, the frequencies of immune cells and their expression of costimulatory markers in VIP-KO mice treated with 7 daily doses of VIPhyb were assayed and were found to be similar to VIP-KO mice treated with PBS (**data not shown**).

### Treatment with Peptide Antagonist of VIP Receptor Enhanced Survival and Reduced Viral Load following mCMV Infection

**Figure 2:**
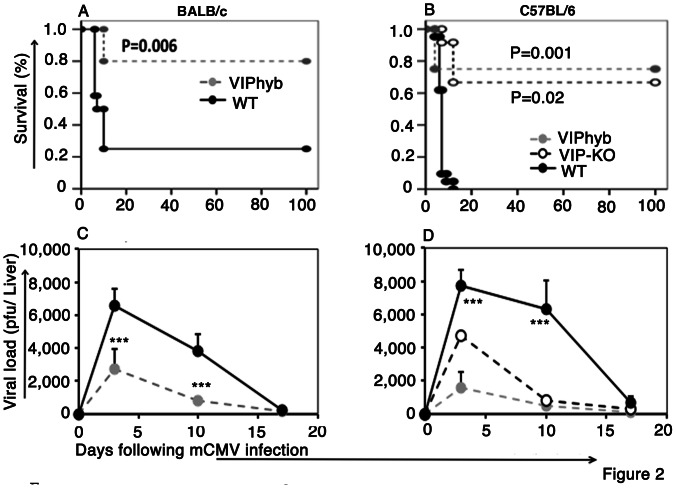
Blocking VIP-signaling improved survival and enhanced viral clearance following mCMV infection. VIP-KO mice and WT littermate with VIPhyb (7 daily subcutaneous doses starting one day before mCMV infection) or WT littermate with PBS were given low-dose 4×10^3^ PFU and high-dose 5×10^4^ PFU (BALB/c), and low-dose 1×10^5^ PFU and high-dose 1×10^6^ PFU mCMV (C57BL/6) by intra-peritoneal injection. Body-weights were checked twice weekly and survival checked daily. Mice receiving lower doses of mCMV were euthanized at 3, 10 and 17 days post infection and liver viral load was measured by plaque assay. A. Survival of BALB/c mice from higher dose mCMV infection. Data are pooled from 2 replicate experiments, n = 10 mice per group. B. Survival of C57BL/6 mice from higher dose of mCMV infection. Data are pooled from 3 replicate experiments, n = 15 mice per group. C. Liver viral load of BALB/c mice from lower dose of mCMV infection (mean ± SEM). Data are pooled from 3 replicate experiments, n = 12 mice per time point and per group. D. Liver viral load of C57BL/6 mice from lower dose of mCMV infection (mean ± SEM). Data are pooled from 5 replicate experiments with a total of 25 mice per experimental group at each time point. *p<0.05, **p<0.01, and ***p<0.001 denote significant differences between VIP-KO mice or VIPhyb-treated mice vs. PBS-treated WT mice.

Next, to explore the effect of administration of VIPhyb on survival following mCMV infection, we compared the effect of treatment with VIPhyb in BALB/c, a mCMV susceptible strain that is Th2 polarized and C57BL/6, a mCMV resistant strain that is Th1 polarized. One day after beginning daily injections of VIPhyb, mice were infected with a lethal dose of mCMV (5×10^4^ PFU for BALB/c mice and 1×10^6^ PFU for C57BL/6 mice). PBS-treated mice had early and rapid mortality following mCMV infection, with only 20% of the BALB/c mice and none of the C57BL/6 mice surviving more than ten days after mCMV infection (**Fig. 2A–B**). In contrast, 80% of the BALB/c mice (**Fig. 2A**), and 75% of the C57BL/6 mice treated with VIPhyb, and 65% of B6 VIP-KO mice (**Fig. 2B**) were long-term survivors. To further explore immune responses to mCMV in VIPhyb-treated mice, we repeated these experiments with a nonlethal dose of mCMV (low-dose PFU) in both BALB/c (sensitive) and C57BL/6 (resistant) strains. Treatment with VIPhyb produced 3–4 - fold reduction of mCMV PFU in the liver (viral load) in both strains of mice compared with control groups treated with PBS (**Fig. 2C–D**).

### Treatment with Peptide Antagonist of VIP Receptor Decreased Inflammation in Liver and Lung of Mice following mCMV Infection

**Figure 3:**
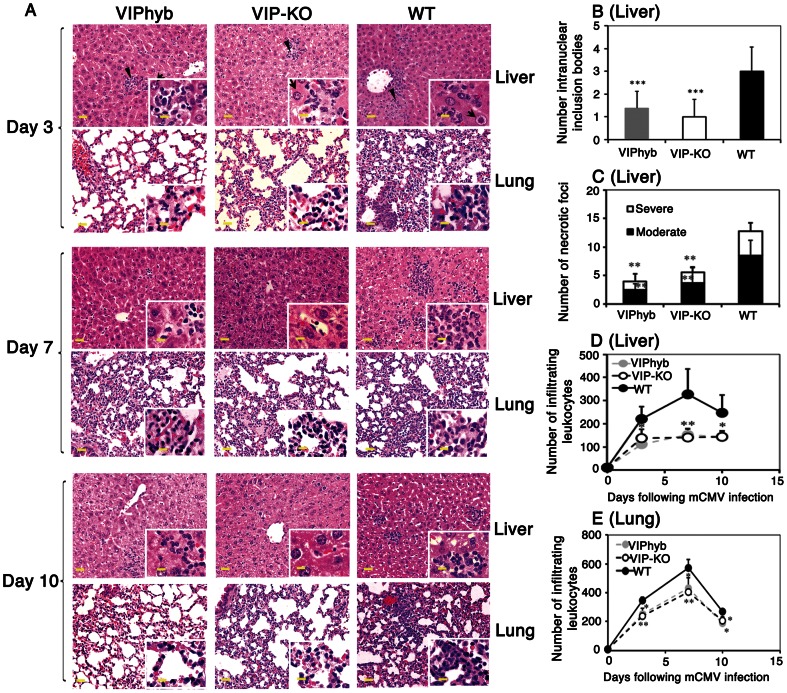
Blocking VIP-signaling decreased inflammation and necrosis in the liver and lung of mCMV-infected mice. VIP-KO and WT littermate C57BL/6 mice treated with 7 daily subcutaneous doses of VIPhyb (starting one day before mCMV infection) or WT littermate with PBS were infected i.p. with 1×10^5^ PFU mCMV. Four mice were euthanized each time-point at 3, 7 and 10 days following mCMV infection. The livers and the lungs were processed for histological analysis. The number of intra-nuclear inclusions (at 20×magnification), numbers of infiltrated nucleated cells, and numbers necrotic foci (at 10×magnification, moderate: necrosis with equal or less than 10 infiltrated nucleated cells, severe: necrosis with more than 10 infiltrated nucleated cells) were determined from hematoxylin and eosin-stained paraffin sections. Data are averaged from 10 random representative area of 0.26 mM^2^ from 2 sections per mouse and per time-point. Data (mean ± SD) are shown from 4 mice per time-point and per group. A: Liver and lung pathology 3,7, and 10 days following mCMV infection. Graphs are shown in 20×magnification with 60×magnification insert. Scale bar (yellow line) is 20 µM. Arrowhead points to necrotic focus; arrow points to intra-nuclear inclusions. B: Number of intra-nuclear inclusions in the liver 3 days following mCMV infection. C: Necrotic foci in the liver 3 days following mCMV infection. D: Infiltrating leukocytes in the liver following mCMV infection. E. Infiltrating leukocytes in the lung following mCMV infection. *p<0.05, **p<0.01, and ***p<0.001 denote significant differences between VIP-KO mice or VIPhyb-treated mice vs. PBS-treated WT mice.

We next tested the effect of blocking VIP-receptor binding on mCMV histopathology in C57BL/6 mice treated with VIPhyb, VIP-KO and control WT mice. Histologic sections of liver and lung following mCMV infection were examined in VIPhyb-treated, VIP-KO, and PBS-treated WT mice (**Fig. 3A**). MCMV infection causes hepatocyte degeneration, necrosis and inflammatory cell infiltrates in the liver [55], [56]. VIPhyb-treated and VIP-KO mice had less inflammation and tissue damage in the liver, with fewer intranuclear viral inclusions, fewer inflammatory leukocytes, less necrosis, and fewer CMV-infected giant cells for 3–10 days after mCMV infection compared with PBS-treated WT mice. (**Fig. 3A–D**). VIP-KO mice and VIPhyb-treated WT mice had similar histological evidence for hepatocyte regeneration at 10 days post-infection as PBS-treated WT mice (**Fig. 3A**), indicating that VIP signaling is not necessary for tissue repair after mCMV infection. Examination of the lungs showed fewer inflammatory leukocytes infiltrating the small peribronchial and inter-alveolar septa following mCMV infection in VIP-KO and VIPhyb-treated mice, as compared with PBS-treated mice (**Fig. 3** **, A and E**).

### VIP Receptor Antagonist Enhanced T-cell Activation

**Figure 4:**
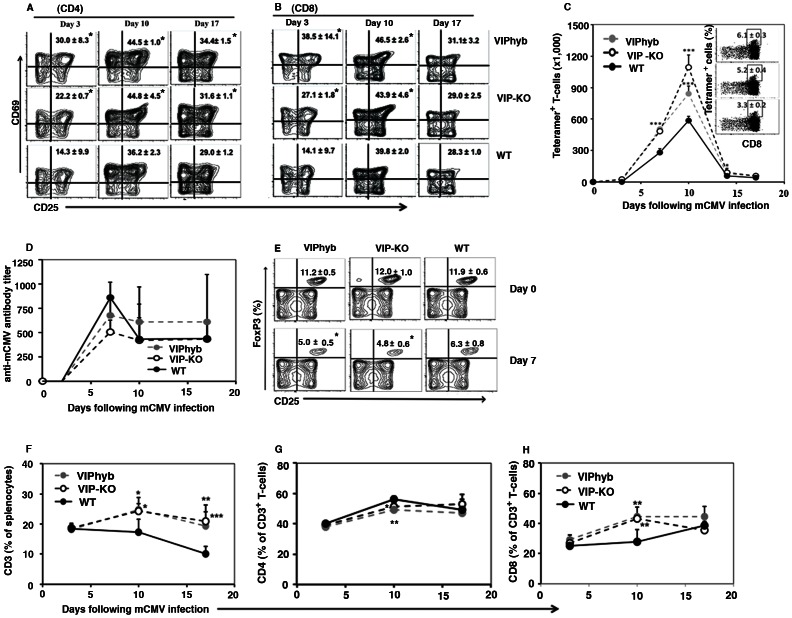
Blocking VIP-signaling increased cellular immune responses following mCMV infection. VIP-KO (black dashed line with open circle) mice or WT littermate C57BL/6 mice treated with short-course of VIPhyb (10 µg/mouse s.c., gray dashed line with filled circle) or PBS (100 µL/mouse s.c., black solid line with filled circle) were infected with 1×10^5^ PFU mCMV day 0 (one day after beginning treatment with VIPhyb or PBS). Mice were euthanized at 3, 10 and 17 days following mCMV infection and the spleens were isolated. Immune cells from the spleens were gated with CD3^+^, CD4^+^ or CD8^+^, and CD62L^−^ and then phenotyped with CD25^+^CD69^+^ and specific anti-viral tetramer^+^ CD8^+^ T-cells by flow cytometry. A: Activated CD4^+^ T-cells. B: Activated CD8^+^ T-cells. Contour plots show data concatenated from 4 mice per time-point and per group, representative of 3 replicate experiments. C. Antigen-specific anti-viral T-cells. Data (mean ± SD, n = 24) summarized from 6 replicate experiments. Inserts: dot plots show data concatenated list mode files of four mice in the same group 7 days following mCMV infection, representative of 6 replicated experiments. D. Serum anti-mCMV antibody levels. Data (mean ± SD, n = 12 per time-point per group) summarized from 3 replicate experiments. E. Percentage of regulatory T-cells in the spleen from C57BL/6 mice pre mCMV infection (day 0) and 7 days following lower dose mCMV infection. Contour plots showed the concatenated list mode files from 5 mice per group 7 days following mCMV infection (mean ± SD), one representative of 2 replicated experiments. F. Percentage of CD3^+^ T-cells in spleen. G. Percentage of CD4^+^ T-cells in spleen. H. Percentage of CD8^+^ T-cells in spleen. *p<0.05, **p<0.01, and ***p<0.001 denote significant differences between VIP-KO mice or VIPhyb-treated mice vs. PBS-treated WT mice.

To explore effect of VIPhyb on activations of lymphocytes in mCMV infection, we tested the activation status of T -cells by measuring expression of early activation markers and numbers of antigen specific CD8^+^ T-cells at serial time points after mCMV infection, using mCMV peptide –(H-2D^b^) MHC class I tetramers [25], [49], [50]. In these experiments only C57BL/6 mice were studied, as the H2D^b^ mCMV-peptide tetramer reagents does not recognize BALB/c T-cells. MCMV infection led to increased numbers of CD62L^−^ CD69^+^ CD25^+^ activated CD4^+^ and CD8^+^ T-cells 3 days following mCMV infection in VIPhyb-treated and VIP-KO mice compared with PBS–treated mice (**Fig. 4** **A–B**). The administration of VIPhyb markedly enhanced the numbers of mCMV tetramer^+^ CD8^+^ T-cells in B6 mice, with kinetics quite similar to what we have seen in mCMV infected VIP-KO mice (**Fig. 4C**) [25]. Peak frequencies of mCMV-peptide-tetramer^+^ CD8^+^ T-cells increased from 584,000±32,000 (mean ± SD) cells per spleen in the WT mice to greater than 860,000±125,000, (p = 0.008) cells per spleen in VIPhyb-treated mice and 1,080,000±125,000, (p<0.001) cells per spleen in the VIP-KO mice (**Fig. 4C**). To test effect of VIPhyb on the activation of B-cells and humoral immunity, serum levels of antibody were analyzed following mCMV infection in C57BL/6 mice. Serum anti-mCMV antibody titers increased in all groups after mCMV infection, but were not significantly different comparing VIP-KO, VIPhyb-treated, and PBS-treated WT mice (**Fig. 4D**), indicating that the effect of VIP-signaling-blockade is limited to enhancement of cellular but not humoral immune responses.

To determine whether enhanced adaptive cellular immunity to mCMV was also associated with decreased regulatory T-cell activity, Treg cells and activated T-cells were measured in the spleen after mCMV infection in C57BL/6 mice. Both VIP-KO and WT mice treated with VIPhyb had fewer splenic Treg 7 days following mCMV infection compared with PBS-treated WT mice (**Fig. 4E–F**). The percentages of splenic CD3^+^ T-cells and CD8^+^ T-cells were increased following mCMV infection in VIP-KO mice and VIPhyb-treated WT mice compared with PBS-treated WT mice for up to 17 days following mCMV infection (**Fig. 4** **, F and H**), while the percentages of CD4^+^ T-cells were decreased in VIP-KO mice and VIPhyb-treated WT mice compared with PBS-treated WT mice 10 days following mCMV infection **(** **Fig. 4G** **)**. To differentiate between increased T-cell proliferation versus increased survival, we measured Ki-67 expression on immune cells. Ki-67 expression on blood CD4, CD8 T-cells and NK cells was not different comparing VIP-KO, VIPhyb-treated, and WT mice 3 days after mCMV infection (**data not shown**). These data suggested that the accumulation of higher numbers of cellular effectors in the absence of VIP signaling might be due to enhanced survival rather than increased proliferation of the activated lymphocytes in the setting of mCMV infection.

To further explore the function of immune cells following mCMV infection, the specific cytolytic activity of immune cells were measured using an in vivo assay in which mCMV peptide pulsed targets are administered to immune mice and the fraction of targets recovered in the spleen measured 16 hours later. VIP-KO and VIPhyb-treated mice had increased cytolytic activities compared with PBS-treated WT mice (**Fig. S1**) [25].

### Treatment with the VIP Receptor Antagonist Enhanced Expression of Costimulatory Molecules and Decreased Expression of Coinhibitory Molecules on T-cells Following mCMV Infection

**Figure 5:**
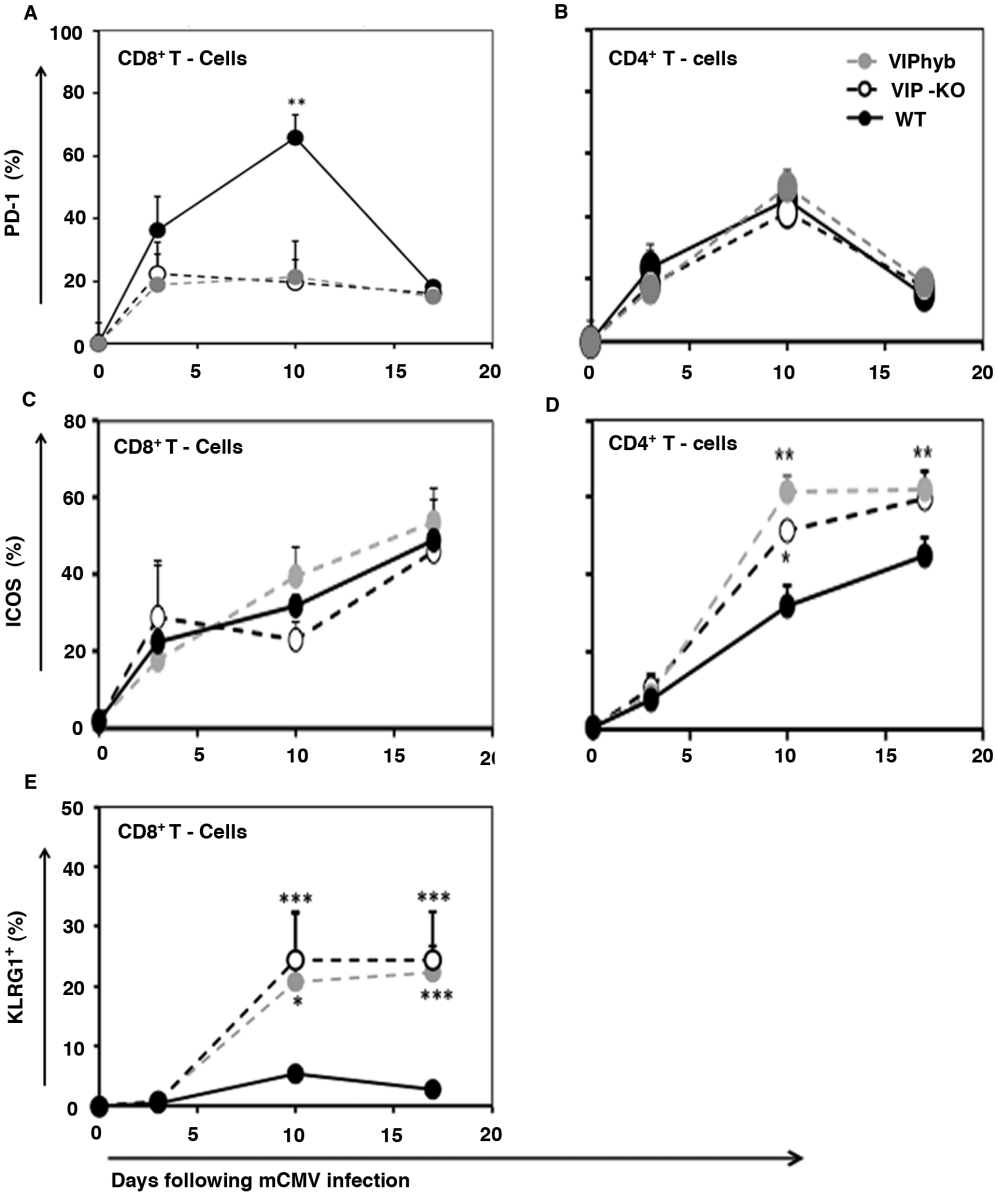
Blocking VIP-signaling increased expression of costimulatory molecules and decreased expression of coinhibitory molecules on lymphocytes following mCMV infection. VIP-KO mice and WT C57BL/6 littermates with 7 daily subcutaneous injections of VIPhyb (starting one day before mCMV infection) or WT littermates treated with PBS were infected i.p. with 1×10^5^ PFU mCMV. Four mice per group were euthanized each time-point at 0, 3, 7, 10 and 17 days post-mCMV infection. The spleens were isolated and phenotypes of activated T-cells and NK cells analyzed by flow cytometry. Data summarized from 3–4 replicate experiments. PD-1 expression on activated CD8^+^ (A) and CD4^+^ T-cells (B) following mCMV infection. Data (mean ± SD, n = 12) summarized from 3 replicate experiments. ICOS expression on activated CD8^+^ (C) and CD4^+^ (D) T-cells following mCMV infection. Data (mean ± SD, n = 12) summarized from 3 replicate experiments. Effector/memory phenotype CD8^+^ T-cells (E). Data (mean ± SD, n = 16) summarized from 4 replicate experiments. *p<0.05, **p<0.01, ***p<0.001 denote significant difference compared with WT treated with PBS group.

To address the mechanism by which treatment with VIPhyb enhanced accumulation of antigen specific T-cells, we next examined the expression of PD-1 and ICOS on activated CD8^+^ and CD4^+^ T-cells. Following mCMV infection, high levels of PD-1 expression were induced in activated CD8^+^ T-cells in WT C57BL/6 mice with the percentage of CD62L^−^CD25^+^CD69^+^ CD8^+^ T-cells expressing PD-1 increasing from 0% at baseline to over 60% ten days after mCMV infection (**Fig. 5A**). Up regulation of PD-1 was nearly completely blocked in C57BL/6 VIP-KO mice and VIPhyb-treated WT C57BL/6 mice (**Fig. 5A**). In contrast, there was no difference in the kinetics of PD-1 expression in activated CD4^+^ T-cells recovered from mCMV infected WT, VIP-KO, or VIPhyb-treated mice (**Fig. 5B**). The expression of ICOS was not effected by blockade of VIP signaling in activated CD8^+^ T-cells, but was up-regulated in CD4^+^ T-cells in VIP-KO mice and VIPhyb-treated mice compared with CD4^+^ T-cells from WT mice (**Fig. 5C–D**). Furthermore, CD62L^−^ CD8^+^ T-cells from VIPhyb-treated mice and VIP-KO mice expressed higher levels of KLRG1 compared with T-cells from WT mice (**Fig. 5E**).

### VIP Receptor Antagonist Enhanced Maturation of DC Following mCMV Infection

**Figure 6:**
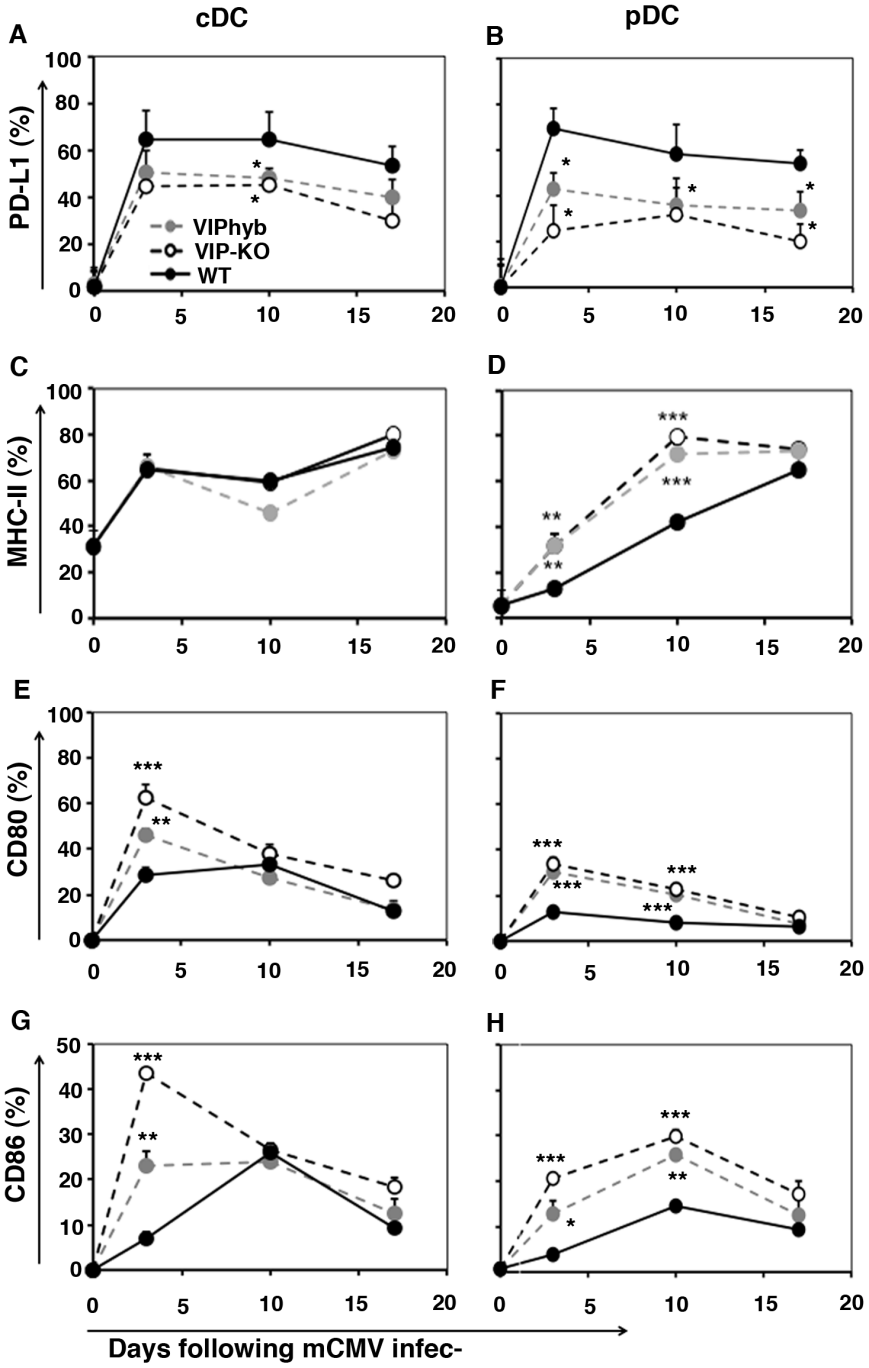
VIP blockade enhanced expression of co-stimulatory molecules and inhibited expression of co-inhibitory molecules on DC following mCMV infection. VIP-KO mice and WT C57BL/6 littermates with 7 daily subcutaneous injections of VIPhyb (starting one day before mCMV infection) or WT littermates treated with PBS were infected i.p. 1×10^5^ mCMV. Mice were euthanized at 0, 3, 10 and 17 days post-mCMV infection and expression of costimulatory molecules on splenocytes were measured by flow cytometry. Data (mean ± SD, n = 12) summarized from 3 replicate experiments. PD-L1 on conventional dendritic cells (cDC, A) and plasmacytoid dendritic cells (pDC, B); MHC-II on cDC © and pDC (D); CD80 on cDC (E) and pDC (F); CD86 on cDC (G) and pDC (H); *p<0.05, **p<0.01, and ***p<0.001 denote significant difference compared with WT mice treated with PBS.

Since mCMV infection up regulates expression of PD-L1 [57] and PD-1/PD-L1 interactions cause immune tolerance that dampen CD8^+^ T-cell anti-viral immune responses in chronic viral infections [58] and, we next measured expression of PD-L1, MHC Class II, CD80 and CD86 on DC subsets isolated at serial time points following mCMV infection in C57BL/6 mice. We found that the percentage of conventional DCs (cDC) and plasmacytoid DCs (pDC) expressing PD-L1 increased from 20% to over 60% within three days of mCMV infection in untreated WT mice (**Fig. 6A–B**). Significantly, the augmentation of PD-L1 expression on both cDC and pDC was markedly attenuated in VIP-KO mice and in WT mice treated with VIPhyb (**Fig. 6C–D**). In addition, while MHC class II expression on cDC was not affected by exposure to VIPhyb, MHC class II expression was enhanced on pDC from CMV-infected VIP-KO mice and VIPhyb-treated mice (**Fig. 6E–F**). In contrast to the suppressive effects of VIP receptor antagonist on PD-L1 signaling, we noted enhanced expression of the co-stimulatory molecules CD80 and CD86 on cDC and pDC in VIP -KO and VIPhyb-treated recipients of mCMV infection (**Fig. 6** **G–H**).

### VIP Receptor Antagonist Treatment Enhanced Levels of Stimulatory Cytokines in NK Cell and T-cells Following mCMV Infection

**Figure 7:**
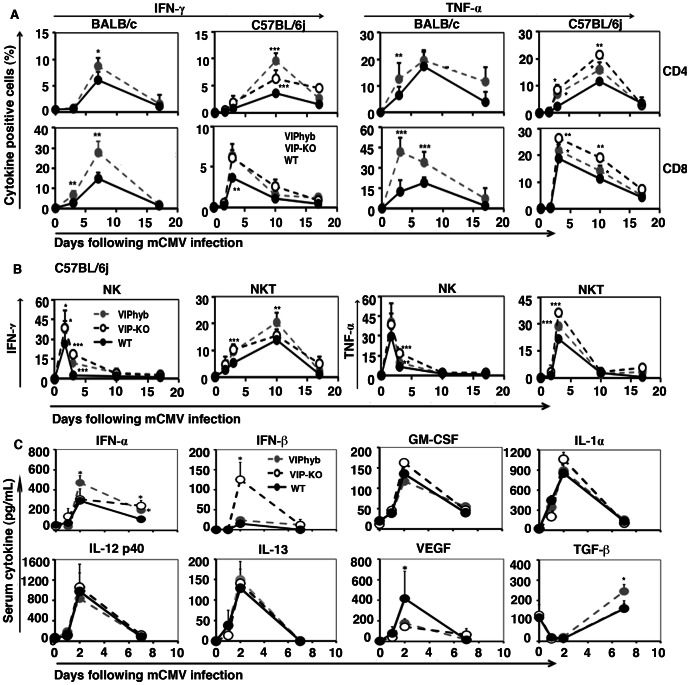
Blocking VIP-signaling enhanced synthesis of inflammatory cytokines following mCMV infection. VIP-KO mice, and WT mice treated with 7 daily s.c. injections of either VIPhyb or PBS and infected (day 0) with low dose mCMV. Four mice were euthanized each time-point at 0, 1, 1.7 (40 hrs), 2, 3, 7, 10 and 17 days following mCMV infection. The spleens were isolated and NK, NK-T, CD4^+^ and CD8^+^ T-cell activation were analyzed by intracellular cytokine expression by flow cytometry. Serum cytokines were analyzed by the Luminex assay. (A) IFN-γ and TNF-α expression on activated CD4^+^ T-cells, and CD8^+^ T-cells from BALB/c and C57BL/6 mice following mCMV infection. Data (mean ± SD, n = 12) summarized from 3 replicated experiments. (B) IFN-γ and TNF-α expression on activated NK cells and NK-T cells from C57BL/6 mice following mCMV infection. (C) IFN-α, IFN-β, GM-CSF, IL-1α, IL-12p40, IL-13, VEGF and TGF-β levels in serum from C57BL/6 mice following mCMV infection. Data (mean ± SD, n = 10) summarized from 2 replicate experiments. *Denotes a significant difference compared with WT mice treated with PBS at that day, p<0.05; **denotes a significant difference compared with WT mice treated with PBS at that day, p<0.01; ***denotes a significant difference compared with WT mice treated with PBS at that day, p<0.001.

To confirm the effect of blocking VIP signaling on immune functions, we measured serum cytokine and levels of intracellular cytokine expression in CD4^+^ and CD8^+^ T-cell, NK T-cells, and NK cell subsets in mice following mCMV infection. In these experiments we compared effects of blocking VIP-signaling in both B6 and BALB/c strains to test the effects on immune polarization in Th1 and Th2 polarized strains, respectively. We were particularly interested in comparing the effect of blocking VIP signaling on the innate immunity of NK cells, as C57BL/6 NK cells are Ly49H^+^ and activated by the m157 epitope while NK cells from BALB/c mice are Ly49H^−^ [46], [47]. BALB/c mice treated with VIPhyb had significantly more interferon-gamma (IFN-γ)^+^ CD4^+^ T-cells seven days after mCMV infection compared with PBS-treated WT BALB/c mice, but the kinetics of induction of IFN-γ was slower than C57BL/6 mice treated with VIPhyb, which had increased IFN-γ^+^ CD8^+^ T- cells 3 days after mCMV infection compared with PBS-treated WT mice (**Fig. 7A**). Intracellular levels of TNF-α expression in CD4^+^ T-cell were marked increased in both BALB/c mice and C57BL/6 mice following treatment of WT mice with VIPhyb (**Fig. 7A**). VIPhyb-treated BALB/c mice had 2–3 fold more TNF-α expressing CD8^+^ T-cells than PBS-treated BALB/c mice (**Fig. 7A**), while an effect on TNF-α expression was not seen in VIPhyb-treated C57BL/6 mice following mCMV-infection. Enhanced numbers of IFN-γ^+^ and TNF-α^+^ expressing NK and NK T-cells were seen in VIPhyb-treated WT and VIP-KO mice, with peak numbers of IFN-γ^+^ NK cells and TNF-α^+^ NK cells seen 40 hours after mCMV infection, compared with PBS-treated WT mice (**Fig. 7B**). NK cells (CD62L^−^NK1.1^+^ CD3^−^) from VIPhyb-treated mice and VIP-KO mice expressed higher levels of KLRG1 a maturation/activation marker compared with NK cells from WT mice (**Fig. S2**).

Finally, since VIP-KO mice retain PACAP, and both VIP and PACAP bind to the VIP receptor, we blocked PACAP binding in VIP-KO mice with seven daily injections of VIPhyb following mCMV infection. VIPhyb-treatment had no additional effect on the enhanced expression of inflammatory cytokines in T-cells and NK cells following mCMV infection compared with PBS-treated control VIP-KO mice (**data not shown**).

To further explore the effect of blocking VIP signaling on immune functions, we measured levels of inflammatory and inhibitory cytokines in serum following mCMV infection in the Th1-polarized C57BL/6 strain. Serum levels of IFN-α and IFN-β in B6 mice were significantly increased 2 days following mCMV infection in mice in which VIP-signaling was blocked (**Fig. 7C**). In contrast, serum levels of GM-CSF, as well as IL-1α, IL-12, IL-13, TGF-β, and chemokines (**Fig. 7C** and **data not shown**), were not significantly changed by VIP-receptor blockade. However, VEGF levels, a molecule associated with immune-suppression [59], [60], were significantly decreased in VIP-KO mice and mice treated with the VIPhyb compared with WT-mice control (**Fig. 7C**).

## Discussion

We have previously reported that VIP-KO mice had enhanced primary and secondary anti-viral cellular immune responses to mCMV infection [25]. In order to further clarify the specific role of VIP in immunity to mCMV infection and facilitate clinical translation, we tested the effect of administering a small peptide antagonist to VIP after mCMV infection. The VIPhyb antagonist is predicted to inhibit VIP, PHI, and PACAP binding to all of the VIP receptors: VPAC1, VPAC2, and PAC1, resulting in reduced down-stream signaling through cAMP/PKA, PLC/PKC, and p38 MAPK pathways. The present data demonstrate that pharmacologic antagonism of VIP/PACAP receptors enhanced cellular immune responses to mCMV infection, accelerating viral clearance, reducing end organ inflammation, and improving survival in both mCMV resistant and susceptible mouse strains. The effect of VIPhyb on the survival and viral load of CMV-infected mice was quite similar to what we have observed in VIP-KO mice, mice that received syngeneic transplants from VIP-KO donors [25], and in VIPhyb-treated B10BR mice following allogeneic hematopoietic stem cell transplants from C57BL/6 donors [26], indicating that pharmacological inhibition of VIP binding is effective in a variety of strains and recapitulates the resistance to mCMV infection seen in VIP-KO mice.

Administering a short-course of a VIPhyb to uninfected mice or to mice infected with mCMV did not produce any discernible toxicity. While Gozes reported that long-term administration of a relatively high dose of VIPhyb antagonist inhibited fetal brain development [45], behavioral effects of treatment with a 7-day course of VIPhyb were not seen in our study. The absence of clinical toxicity in the present study may be due to the fact that we used lower doses of VIPhyb for a short period [43] in adult mice with more developed blood-brain barriers [61]. Of note, brain pathology of VIPhyb-treated mice was not examined in this study and therefore, neuro-pathologic effects of VIPhyb cannot be completely excluded. While published data showed that VIP suppressed bone marrow hematopoiesis through VPAC1 [62], mice treated with VIPhyb and VIP-KO mice had similar numbers of leukocytes in the blood and bone marrow, similar serum levels of GM-CSF, and similar numbers of hematopoietic cell populations as PBS-treated WT mice, indicating a limited effect of VIPhyb on hematopoiesis.

The mechanism by which VIP-signaling blockade enhances immune responses is complex. In immune cells, VIP binding to its receptors initiates a cascade of signaling that limits activation of the innate and adaptive immune systems. Physiological VIP-signaling in response to inflammatory signals leads to increased cAMP and enhanced immune suppressive activity of Treg, with decreased T-cell proliferation and IL-2 production [63]. VIP, as well as the closely related neuropeptide PHI and PACAP, binds to three known GPCR receptors- VPAC1, VPAC2, and PAC1. T-cells and DC express VPAC1 and VPAC2, but not PAC1 [27]. T-cell activation and differentiation induces VPAC2 expression, while down-regulating VPAC1 expression [64]. The very similar immune effects seen in VIPhyb-treated mice (in which VPAC1, VPAC2 and PAC1 receptors are blocked) compared with VIP-KO mice (lacking VIP and PHI) suggests that VIP (or PHI) signaling to VPAC1 and/or VPAC2 is most relevant to regulating immune responses in the mCMV infection models. VIP polarizes CD4^+^ T-cells toward an immunosuppressive Th2 response while suppressing the Th1 responses [65] and VIP induces tolerogenic DC, which moderates T-cell activity through increased induction of Treg [37]. Th2 polarization of immune responses by VIP-differentiated DC is likely achieved through down-regulation of costimulatory signals on APC and inhibition of TNF-α, IL-1, IL-6, and IL-12 production following VIP-signaling through VPAC1 and VPAC2 [66]. Of note, increased VEGF expression seen in WT BL/6 mice after mCMV infection but not in VIPhyb-treated or VIP-KO mice has been also associated with decreased DC maturation [60] and VIP-signaling [67], [68]. Blockade of VIP-receptor interactions directly enhanced DC maturation and activated NK cells, and then stimulated adaptive T-cell immune responses to mCMV. Of note, plasmacytoid DC (pDC) are the major source of type one interferon following viral infections and are most relevant to the initiation of adaptive immune responses, while classical DC (cDC) are more relevant to continued antigen presentation to activated T-cells. We observed a striking effect of enhanced co-stimulatory molecule and MHC II expression on both pDC and cDC with VIP receptor blockade and increased serum levels of type-I IFN (IFN-α and IFN-β) indicating effects on both DC populations. CD8+ T-cells are the major effector cell population involving in killing virally infected cells while CD4+ T cells augment and sustain adaptive immune responses through cytokine production. Blocking the VIP receptor and genetic deletion of VIP/PHI led to decreased PD-1 expression on effector CD8+ T-cells and increased ICOS-L expression on CD4+ T-cells as well as augmentation of expression of Th1 cytokines (IFN-γ and TNF-α) on day 3 post-infection in CD8+ T-cells and on day 10 in CD4+ T-cells. Taken together, these data are consistent with the absence of VIP binding or signaling leading to a generalized activation of adaptive cellular immunity following mCMV infection.

The increased numbers of anti-viral CD8^+^ T-cells seen in the absence of VIP binding and signaling was not mainly due to the proliferation of T-cells, but to their increased accumulation, concomitant with down-regulation of PD-L1 and PD-1 expression on DCs and activated CD8^+^ T-cells, respectively. The temporal relationship between decreased PD-L1/PD-1 expression and enhanced numbers of anti-viral CD8^+^ T-cells indicate that the effect of down-regulating PD-1/PD-L1 expression in the absence of VIP binding and signaling had a positive effect on the magnitude and persistence of anti-viral immune responses. Up-regulation of PD-1 expression on activated T-cells [69], [70], has been shown following acute [58] and chronic [71] viral infections and is associated with decreased antiviral immunity, a phenomenon termed immunological “exhaustion” [71]. Increased numbers of CD62L^−^KLRG1^+^ effector/memory CD8^+^ T-cells and mature NK cells seen with VIP-signaling blockade confirmed that inhibiting VIP binding increased and prolonged the activity and persistence of effector/memory T-cells [58] and mature/memory NK cells [72], [73].

In contrast to the Th1-polarized inflammatory effects seen when VIP binding is inhibited, intact VIP-signaling leads to Th2 immune polarization, favoring humoral immunity over Th1 cellular-mediated immune polarization [39], [74]. We expected that blockade of VIP-signaling would suppress humoral immunity due to decreased Th2 polarization. Surprisingly, we found that blockade of VIP signaling had minimal effects on the kinetics of anti-viral antibodies comparing WT control mice to VIP-KO mice and VIPhyb-treated mice following mCMV infection. As expected, the peak humoral immune responses, as assessed by anti-mCMV antibody titers, occurred later than the peak numbers of activated NK cells and antiviral T-cells. The preservation of humoral immunity seen with VIPhyb antagonism may be due to increased IFN-γ production by NK and T-cells [75], [76] and enhanced ICOS expression on CD4^+^ T-cells [77], [78], two signaling pathways promote antibody synthesis by B-cells.

VIPhyb antagonism slightly reduced the percentage of Treg seen in the spleen after mCMV infection. PD-1 and PD-L1 signaling and VEGF promote generation of Treg cells [79] [59], [80], and Treg are induced in vivo following exposure to exogenous VIP [38]. Our data show that the decreased numbers of Treg cells were directly correlated with increase of pro-inflammatory cytokines, reduced VEGF and decreased expression of PD-1 and PD-L1 following mCMV infection, indicating that blocking VIP binding and signaling induces a global effect on immune checkpoints following an inflammatory stimulus.

The peak plasma levels of VIPhyb following daily administration of 10 µg peptide are predicted to be low, around 0.6 µM, or about 1/10 of the reported IC50 for antagonism ^125^I-VIP binding to cells expressing VPAC1. Nonetheless, we observed a strong enhancement of antiviral immunity following daily treatment with VIPhyb. While VIPhyb is 10-fold less potent in antagonizing VIP binding than another synthetic VIP peptide derivative (SN-VIPhyb), and 100 fold less potent than VIP(3–7)/GRF(8–27), VIPhyb was selected for the current studies based upon its ability to inhibit cAMP concentration in VIPhyb-treated cells, and the reported lack of toxicity in mice. Our data suggest that partial or transient blockade of the VIP receptor may sufficient to regulate immune checkpoints.

In conclusion, the administration of VIPhyb transiently activates cellular immunity following murine CMV infection. Given the homology between human and murine CMV, and the sequence identity between murine and human VIP [81], the clinical use of a VIP antagonist may be a novel approach to enhance innate and adaptive cellular immunity to vaccines and viral infection in patients. Further studies are needed to understand the antiviral activity of VIPhyb including studies of selective VPAC1 and VPAC2 antagonists and optimization of the doses and schedule of VIP receptor antagonists.

## Supporting Information

Figure S1 **VIPhyb-treated mice and VIP-KO mice had increased cytolytic activity against M45 peptide-pulsed targets following mCMV infection.** A mixture of peptide-pulsed targets (CD45.1^+^ CD45.2^+^) and non-pulsed targets (CD45.2^−^ CD45.1^+^) were adoptively transferred to VIP-KO,VIPhyb-treated and PBS-treated WT mice 9 days after infection with low-dose mCMV. Target cells were harvested from the recipient spleens 16 hours after iv injection, and peptide-pulsed targets and non-pulsed targets were differentiated by flow cytometry following staining for CD45.1^+^ and CD45.2^+^ cells, respectively.The data shown calculated mean specific cytolytic activity from two replicate experiments (n = 10).
(TIFF)

Figure S2 **Blocking VIP-signaling increased maturation/memory phenotype of NK following mCMV infection.** VIP-KO mice and WT C57BL/6 littermates with 7 daily subcutaneous injections of VIPhyb (starting one day before mCMV infection) or WT littermates treated with PBS were infected i.p. with 1 × 105 PFU mCMV. Four mice per group were euthanized each time-point at 0, 3, 7, 10 and 17 days post-mCMV infection. The spleens were isolated and phenotypes of NK cells analyzed by flow cytometry. Data summarized from 3–4 replicate experiments. The percentage of KLRG1+ NK cells are shown. Data (mean ± SD, n = 16) summarized from 4 replicate experiments. *p<0.05, ***p<0.001 denote significant difference compared with WT treated with PBS group.
(TIFF)
