# Supplementary figures and images for: VIPhyb, an Antagonist of Vasoactive Intestinal Peptide Receptor, Enhances Cellular Antiviral Immunity in Murine Cytomegalovirus Infected Mice
Source: PLoS One. 2013 May 27;8(5):e63381. doi: 10.1371/journal.pone.0063381 (PMC3664580; doi:10.1371/journal.pone.0063381)

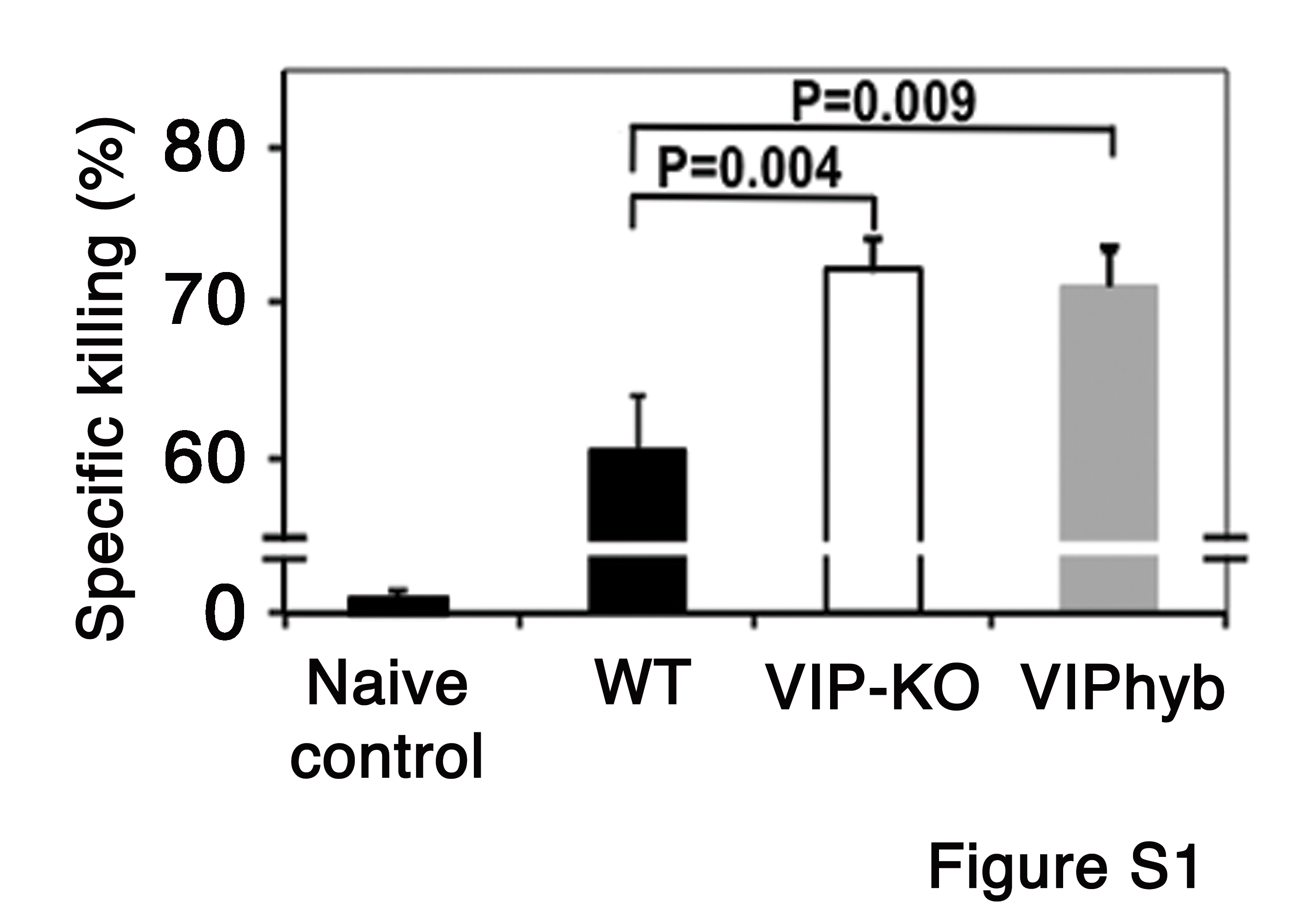

Supplement: Figure S1 — VIPhyb-treated mice and VIP-KO mice had increased cytolytic activity against M45 peptide-pulsed targets following mCMV infection. A mixture of peptide-pulsed targets (CD45.1+ CD45.2+) and non-pulsed targets (CD45.2− CD45.1+) were adoptively transferred to VIP-KO,VIPhyb-treated and PBS-treated WT mice 9 days after infection with low-dose mCMV. Target cells were harvested from the recipient spleens 16 hours after iv injection, and peptide-pulsed targets and non-pulsed targets were differentiated by flow cytometry following staining for CD45.1+ and CD45.2+ cells, respectively.The data shown calculated mean specific cytolytic activity from two replicate experiments (n = 10). (TIFF) [file pone.0063381.s001.tiff]

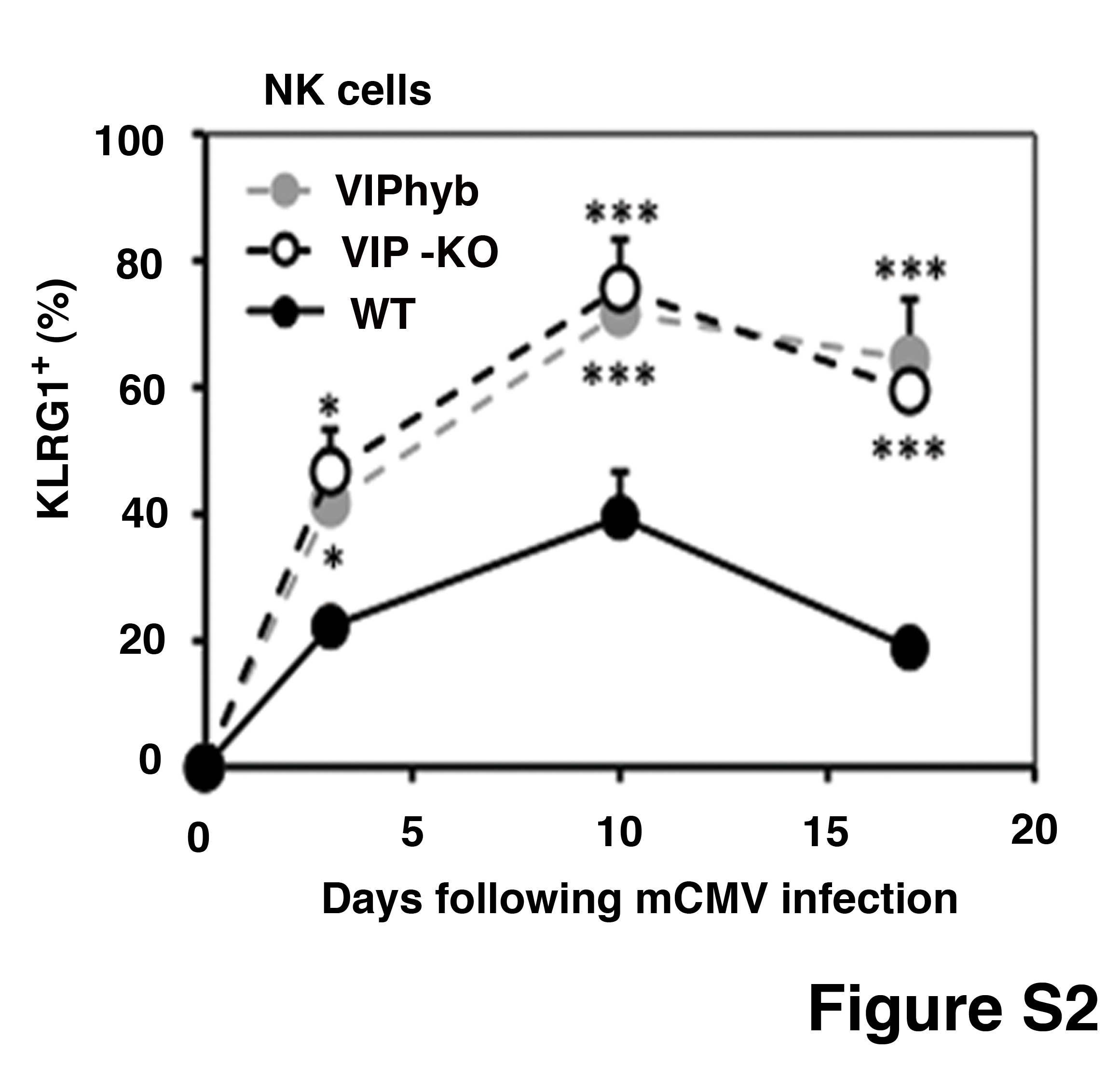

Supplement: Figure S2 — Blocking VIP-signaling increased maturation/memory phenotype of NK following mCMV infection. VIP-KO mice and WT C57BL/6 littermates with 7 daily subcutaneous injections of VIPhyb (starting one day before mCMV infection) or WT littermates treated with PBS were infected i.p. with 1 × 10^5^ PFU mCMV. Four mice per group were euthanized each time-point at 0, 3, 7, 10 and 17 days post-mCMV infection. The spleens were isolated and phenotypes of NK cells analyzed by flow cytometry. Data summarized from 3–4 replicate experiments. The percentage of KLRG1+ NK cells are shown. Data (mean ± SD, n = 16) summarized from 4 replicate experiments. *p<0.05, ***p<0.001 denote significant difference compared with WT treated with PBS group. (TIFF) [file pone.0063381.s002.tiff]
